# Acrolein Aggravates Secondary Brain Injury After Intracerebral Hemorrhage Through Drp1-Mediated Mitochondrial Oxidative Damage in Mice

**DOI:** 10.1007/s12264-020-00505-7

**Published:** 2020-05-21

**Authors:** Xun Wu, Wenxing Cui, Wei Guo, Haixiao Liu, Jianing Luo, Lei Zhao, Hao Guo, Longlong Zheng, Hao Bai, Dayun Feng, Yan Qu

**Affiliations:** Department of Neurosurgery, Tangdu Hospital, Fourth Military Medical University, Xi’an, 710038 China

**Keywords:** Intracerebral hemorrhage, Secondary brain injury, Acrolein, Drp1, Mitochondrial oxidative damage

## Abstract

Clinical advances in the treatment of intracranial hemorrhage (ICH) are restricted by the incomplete understanding of the molecular mechanisms contributing to secondary brain injury. Acrolein is a highly active unsaturated aldehyde which has been implicated in many nervous system diseases. Our results indicated a significant increase in the level of acrolein after ICH in mouse brain. In primary neurons, acrolein induced an increase in mitochondrial fragmentation, loss of mitochondrial membrane potential, generation of reactive oxidative species, and release of mitochondrial cytochrome c. Mechanistically, acrolein facilitated the translocation of dynamin-related protein1 (Drp1) from the cytoplasm onto the mitochondrial membrane and led to excessive mitochondrial fission. Further studies found that treatment with hydralazine (an acrolein scavenger) significantly reversed Drp1 translocation and the morphological damage of mitochondria after ICH. In parallel, the neural apoptosis, brain edema, and neurological functional deficits induced by ICH were also remarkably alleviated. In conclusion, our results identify acrolein as an important contributor to the secondary brain injury following ICH. Meanwhile, we uncovered a novel mechanism by which Drp1-mediated mitochondrial oxidative damage is involved in acrolein-induced brain injury.

**Electronic supplementary material:**

The online version of this article (10.1007/s12264-020-00505-7) contains supplementary material, which is available to authorized users.

## Introduction

Intracerebral hemorrhage (ICH) is an acute cerebrovascular event with a high mortality and disability rate [1, 2]. Pathological processes after ICH include not only primary brain injury, but also secondary brain injury (SBI) [3, 4]. Primary brain injury refers to the physical disruption of the cellular architecture induced by the initial hematoma. Meanwhile, the hematoma leads to SBI, which includes oxidative damage, the inflammatory response, glutamate toxicity, neural death, and blood-brain barrier disruption [5–7]. Strategies targeting SBI are of great importance for both experimental and clinical studies. Although multiple treatments have emerged in clinical practice, the outcomes of ICH remain unsatisfactory [8]. These considerations impelled us to explore whether there are critical pathological factors that have not been focused on in previous treatments.

Acrolein (CH2=CH–CHO) is a highly active unsaturated aldehyde [9] that is more reactive than 4-hydroxynonenal and is closely involved in tissue damage [10, 11]. Due to its high reactivity and electrophilic activity, acrolein is capable of inducing cellular oxidative injury, damaging the redox balance, and resulting in cell death [12–14]. Acrolein-induced toxicity has been implicated in many nervous system diseases. Previous studies reported a remarkable increase in acrolein level in the brain tissue of parkinsonian sufferers [15] as well as after acute spinal cord injury [16]. And an increased plasma level of acrolein was found to be a good biomarker for brain infarction size in cerebral ischemic stroke patients with high sensitivity and specificity [17]. However, so far, the role it plays in secondary injury following ICH is not clear.

Mitochondrial quality-control has been demonstrated to play an essential role in protecting against ICH injury [18, 19]. Mitochondria, highly dynamic organelles, constantly undergo fission and fusion to maintain their normal morphology and function [20, 21]. Aberrant modulation of mitochondrial dynamics, which shifts the balance of fusion and fission towards fission, participates in the pathological process of hemorrhage-induced acute neural injury [22, 23]. Under physiological conditions, mitochondrial fission is of vital importance in mitochondrial biogenesis and in the removal of impaired mitochondria. However, excessive fission can impair mitochondrial structure, leading to damaged oxidative phosphorylation, increased mROS generation, and shortage of ATP [24, 25]. Mitochondrial fission is mainly regulated by dynamin-related protein 1 (Drp1), a cytoplasmic protein that is recruited to the outer mitochondrial membrane to trigger mitochondria fission [26]. Phosphorylation is a permissive step in the regulation of Drp1 recruitment in different settings. It is generally accepted at present that phosphorylation at serine 616 (S616) of Drp1 accelerates its recruitment to the mitochondrial membrane [27], while phosphorylation at S637 in Drp1 inhibits this process [28, 29].

Acrolein has been shown to result in oxidative stress and promote mitochondrial dysfunction *via* a variety of intra- and inter-cellular signaling mechanisms [12, 30]. However, whether Drp1-mediated mitochondrial fission contributes to acrolein-induced neurotoxicity has not been investigated. In this study, we explored whether acrolein plays a detrimental role in SBI after ICH injury mechanistically, focusing on Drp1-mediated mitochondrial oxidative damage.

## Materials and Methods

### Animals

All experimental procedures described here were approved by the Ethics Committee of the Fourth Military Medical University and strictly followed the guidelines of the National Institutes of Health Guide for the Care and Use of Laboratory Animals. Heathy male C57BL/6 background mice (8 weeks–12 weeks old, 20 g–25 g) were purchased from the Laboratory Animal Centre of the Fourth Military Medical University. They were kept under a regular 12-h light/dark cycle at 18°C–22°C, and had free access to water and food.

### ICH Surgery and Drug Administration

The mouse model of ICH was established by collagenase VII injection as described previously [31]. Each mouse was anesthetized with 2% pentobarbital sodium and the head was fixed. Then we drilled a hole ~0.5 mm in diameter, and a needle was advanced to the corpus striatum under stereotactic guidance (coordinates: 0.20 mm anterior, 2.30 mm right lateral, 3.50 mm deep). Collagenase VII-S (Sigma-Aldrich, St Louis, MO) (0.075 U diluted in 0.25 μL saline) was administered slowly, after which the needle was left for ~5 min to allow full absorption. The sham group underwent the same procedures without collagenase infusion. Hydralazine (MedChemExpress, HY-B0464) was dissolved in phosphate-buffered saline. Mice in the treatment group were intraperitoneally injected with 5 mg/kg hydralazine immediately after the surgery.

### Primary Neuron Culture

Primary neurons were cultured as described previously [32]. Briefly, the corpus striatum was isolated from fetal C57BL/6 mice and digested in 0.125% trypsin for 0.5 h. The suspension was added to a 24-well plate pre-coated with poly-*L*-lysine. Then the cells were maintained in Dulbecco’s modified Eagle’s medium supplemented with 10% fetal bovine serum, 1% penicillin–streptomycin and 1% *L*-glutamate under 5% CO_2_ for 4 h at 37°C. The medium was then substituted with 500 μL Neurobasal medium containing B27, 100 U/mL penicillin, and 100 U/mL streptomycin. After 3 and 7 days, the cells were treated with cytosine arabinoside for 24 h to inhibit glial cell growth. Neurons were ready for experiments on day 14.

### Analysis of Mitochondrial Function

The mitochondrial membrane potential (MMP) was measured using tetramethyl rhodamine ethyl ester (TMRE) staining, and mitochondrial reactive oxygen species (ROS) production using MitoSox Deep Red staining, as previously described [33]. Primary neurons were incubated with 10 nmol/L TMRE (T669, Life Technologies, Carlsbad, CA) or 5 nmol/L MitoSox (M36008, Invitrogen, Carlsbad, CA) for 0.5 h. Then, the cells were washed 3 times with Hanks’ Balanced Salt Solution to remove the medium. Last, images were captured with a fluorescence microscope (A1 Si, Nikon, Tokyo, Japan) and ImageJ (National Institutes of Health, Bethesda, MD) software was used to quantify the relative fluorescence levels.

### Measurement of Brain Water Content

Brain water content was assessed with the wet-dry method as we previously described [34]. The hemispheres with hemorrhage were separated 72 h after ICH and weighed to obtain the wet weight. Then, we put them into an oven (95°C–100°C; 72 h) to obtain the dry weight. Brain water content = [(wet weight − dry weight)/wet weight] × 100 %.

### Neurological Outcomes

We used the modified neurological severity score (mNSS) to assess the degree of neurological functional impairment as described previously [35]. The mNSS, which contains motor, sensory, and reflex tests, ranges in score from 0 to 18 (0 means normal; score 18 means maximal severity). The higher the score, the worse the sensorimotor function. The scoring was carried out on day 3 after ICH by two observers who were blinded to the groups.

### Quantification of Hematoma Volume

The hematoma volume was measured using a method previously described [31]. In brief, ten 10-μm cryosections from the ventral to the dorsal edge of the hematoma at 100-μm intervals were used to quantify the hematoma area using ImageJ software. ICH volume = (hemorrhage area × distance between sections) − (epicenter of hemorrhage area × section thickness). All measurements were performed by two observers who were blind to the experiment.

### Immunofluorescence and TUNEL Staining

We performed immunofluorescence staining as described previously [36]. Briefly, each mouse was sacrificed by an overdose of 2% pentobarbital sodium at 72 h after ICH, followed by trans-cardiac perfusion with 4% paraformaldehyde. The brain was removed, stored in 4% paraformaldehyde overnight, and then dehydrated in 10%, 20%, and 30% sucrose. The brain was then sectioned at 15 μm–25 μm for further experiments and analysis. After treatment with 0.3% Triton X-100 for 30 min, and 10% donkey serum for 2 h, the sections were incubated with the following primary antibodies: mouse anti-acrolein (1:200, Abcam, Cambridge, MA), rabbit anti-NeuN (1:1000, Abcam), chicken anti-GFAP (1:1000, Invitrogen), and rabbit anti-IBA1 (1:1000, Wako, Tokyo, Japan). After incubation at 4°C overnight and 3 × 5-min washes with PBS, they were incubated with the following secondary antibodies: donkey anti-rabbit IgG (Alexa Fluor 594), donkey anti-chicken IgG (Alexa Fluor 594), and donkey anti-mouse IgG (Alexa Fluor 488). Then, the sections were stained with DAPI for 15 min at room temperature. All sections were examined blindly using an A1 Si confocal microscope (Nikon). Representative images were captured from three independent experiments using six different mice.

TUNEL staining was performed according to the manufacturer’s protocol (Roche, Mannheim, Germany). After treatment with 0.3% hydrogen peroxide (30 min) and incubation with 0.25% proteinase K (45 min, 37°C), selected sections were immersed in TUNEL reaction solution in the dark (1 h, 37°C). Last, the sections were stained with DAPI for 10 min (37°C). The extent of apoptosis was evaluated as the ratio of TUNEL-positive cells to DAPI-stained cells. This evaluation was performed blindly. Images were captured from at least three independent experiments using six mice.

### Transmission Electron Microscopy

Transmission electron microscopy was performed as previously described [37]. Briefly, each mouse was sacrificed by anesthetic overdose 72 h after the ICH operation, followed by perfusion with 0.9% saline then 4% paraformaldehyde. The hematoma was trimmed into blocks 1 mm–2 mm wide. After post-fixation in 1% osmium tetroxide for 1 h, the blocks were dehydrated in ethanol, fixed overnight in 4% glutaraldehyde, and embedded in resin. An ultramicrotome was then used to cut the blocks into 80-nm sections. Finally, the ultrathin sections were viewed in a JEM-1400 electron microscope (JEOL, Tokyo, Japan) and micrographs were captured with a charge-coupled device camera (Olympus, Tokyo, Japan).

### Western Blots

We performed western blots according to the procedures described previously [38]. Selected samples were homogenized in lysis buffer containing 1% protease inhibitor. Protein concentrations were measured with a BCA Protein Assay kit (UA276918; Thermo Scientific, Waltham, MA). First, we separated the protein samples on SDS-PAGE gels, followed by transfer to PVDF membranes (Millipore, Billerica, MA). Then the membranes were blocked with 5% non-fat milk diluted in TBST and incubated with primary antibodies overnight at 4 °C. After 3 × 5-min washes in TBST, the membranes were incubated with the corresponding horseradish peroxidase-conjugated secondary antibodies (1:5000, 27°C, 2 h) followed by 3 × 10-min washes in TBST. Finally, we assessed the protein bands with a BioRad imaging system (Bio-Rad, Hercules, CA). The primary antibodies used in the experiments were as follows: acrolein (1:1000, ab240918, Abcam, Cambridge, MA); phospho-DRP1 (Ser637) (1:800, ab193216, Abcam); DRP1 (1:1000, D6C7, Cell Signaling, Boston, MA); phospho-DRP1 (Ser616) (1:1000, D9A1, Cell Signaling); AMPK (1:1000, 2532S, Cell Signaling); phospho-AMPK (1:1000, D4D6D, Cell Signaling); ERK (1:1000, 137F5, Cell Signaling); phospho-ERK (1:1000, 197G2, Cell Signaling); OPA1 (1:1000, D6U6N, Cell Signaling); Mfn2 (1:1000, D2D10, Cell Signaling); Mfn1 (1:1000, A9880, ABclonal, Wuhan, China); Bax (1:1000, gtx32465, Gene Tex, Alton Pkwy Irvine, CA), Bcl2 (1:1000, gtx100064, Gene Tex); cytochrome c (1:1000, wh118104, Wanleibio, Shanghai, China); and β-actin (1:3000, wh096194, Wanleibio).

### Statistical Analysis

Neurological function scores were analyzed with Kruskal–Wallis one-way analysis of variance on ranks, followed by the Student–Newman–Keuls test. Comparisons between two groups were analyzed using Student’s *t* test (unpaired, two-tailed), while comparisons between multiple groups were analyzed using one-way analysis of variance (ANOVA), followed by the Tukey *post hoc* test. *P*-values <0.05 were defined as statistically significant. All data are expressed as the mean ± SEM. The analysis was performed with SPSS (version 21.0, IBM, Chicago, IL).

## Results

### Upregulation of Acrolein in Mouse Brain after ICH

**Fig. 1:**
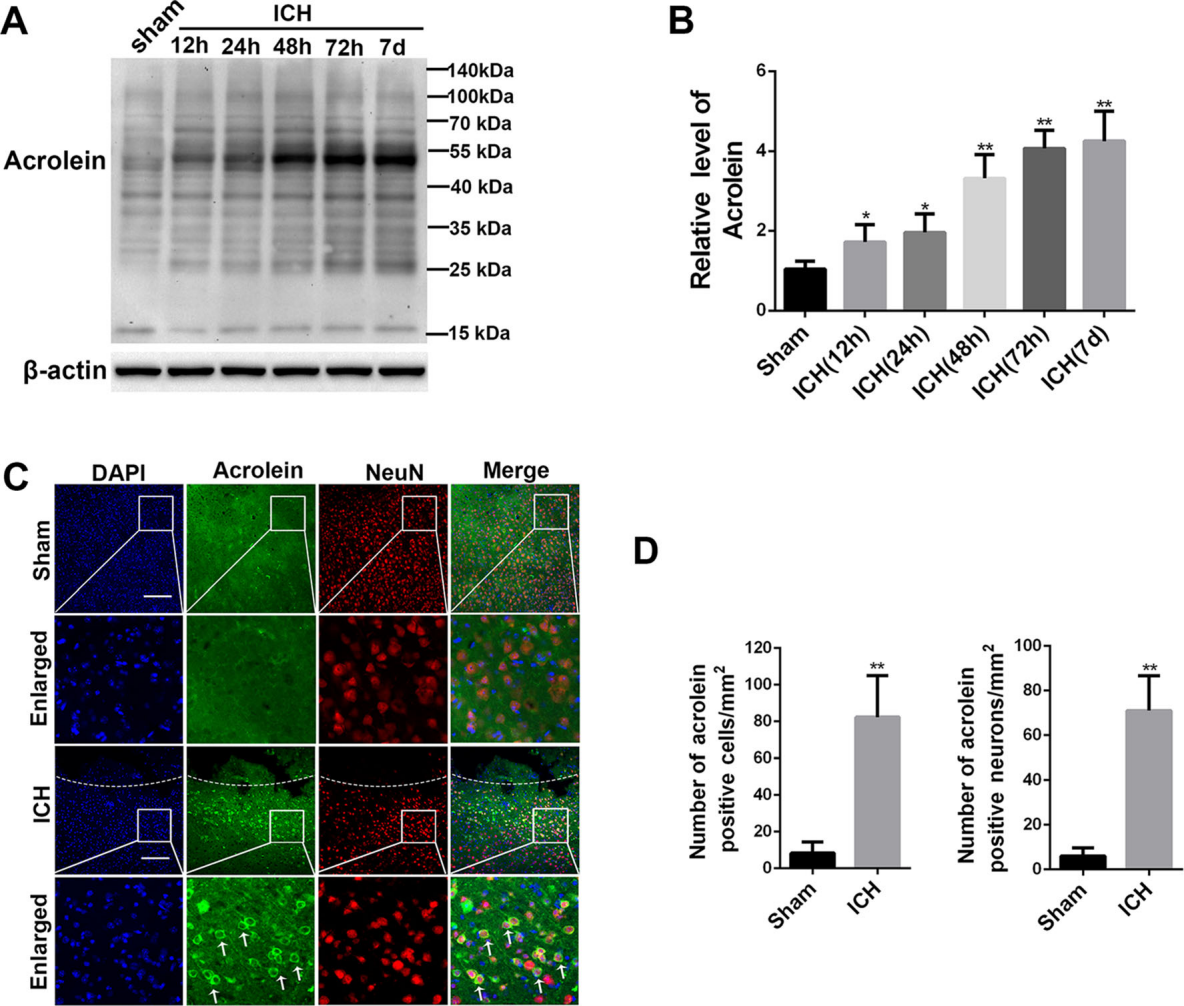
The elevated level of acrolein in mouse brain after ICH. **A, B** Western blots (**A**) and analysis (**B**) of the acrolein levels in peri-hematoma tissue at 12 h, 24 h, 48 h, 72 h, and 7 days after ICH. **C** Representative images of double immunofluorescence staining for acrolein (green) with NeuN (red) in the peri-hematoma area 72 h after ICH (scale bars, 200 μm; arrows indicate acrolein-positive cells; dashed lines mark the hemorrhage sites). **D** Numbers of acrolein-positive cells (left) and neurons (right). *n* = 6 mice per group; data are presented as the mean ± SEM, *P < 0.05, **P < 0.01 *vs* sham group.

First, we used western blotting to explore possible alterations in the acrolein level in peri-hematoma tissues from different groups (sham, 12 h, 24 h, 48 h, 72 h, and 7 days after ICH). The acrolein level was increased at 12 h, reached a peak at 72 h, and remained at a high level to 7 days (Fig. 1A, B). Since the marked elevation of acrolein levels occurred at 72 h post-ICH, this time was adopted as the post-ICH time point for the subsequent experiments. Furthermore, double immunofluorescence staining of sections from 72 h post-ICH mice predominantly revealed an abundant co-location of acrolein with neurons (Fig. 1C), but little co-labeling with astrocytes and microglia (Fig. S1A–C). These results indicated that the level of acrolein is significantly elevated in neurons around the hematoma after ICH in mice.

### Acrolein Induces Morphological Alterations and Functional Impairment in the Mitochondria of Primary Neurons

**Fig. 2:**
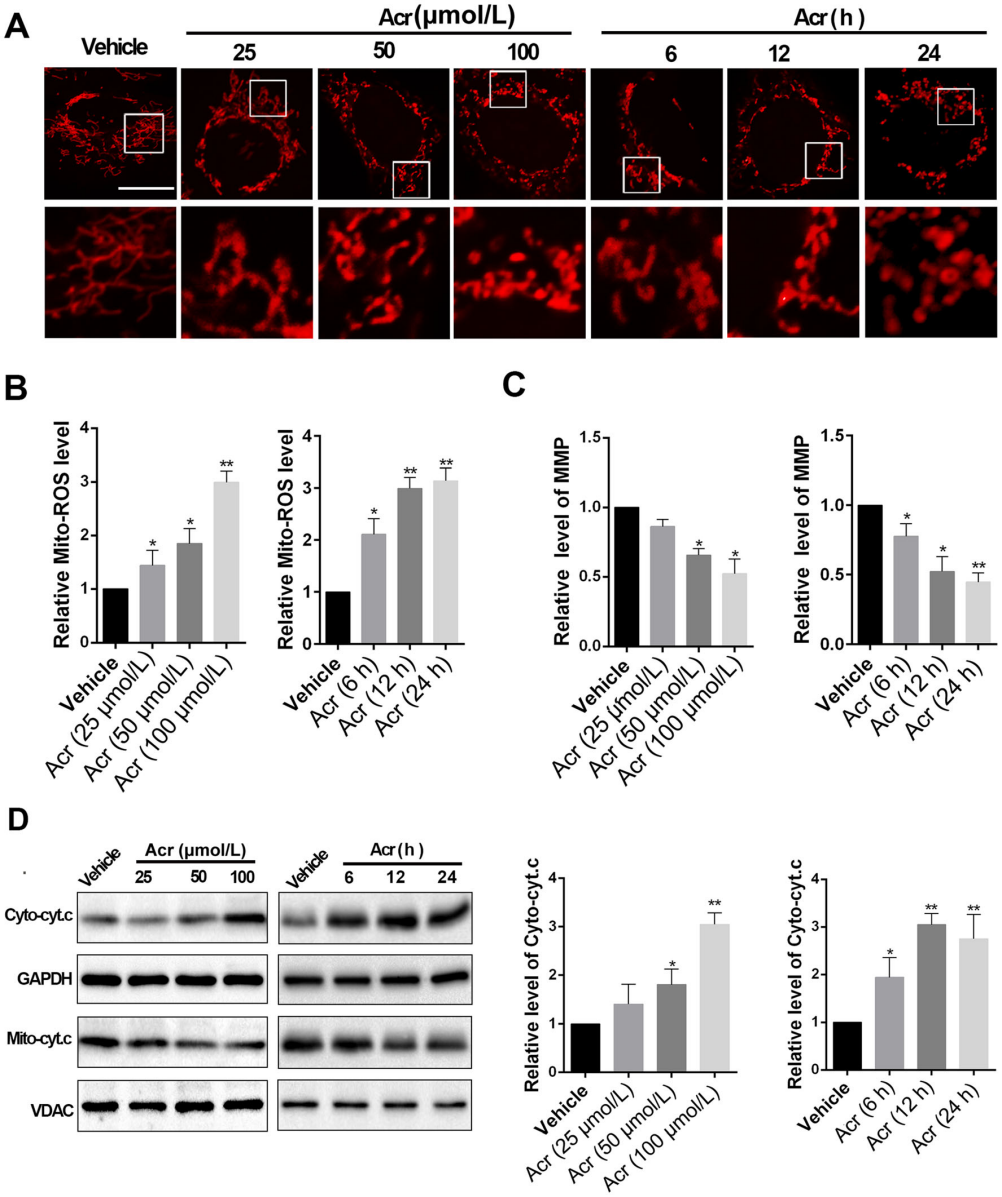
Acrolein induces morphological alterations and functional impairment of mitochondria in primary neurons. Step 1: primary neurons were treated with 25, 50, or 100 μmol/L acrolein for 12 h. Step 2: primary neurons were treated with 100 μmol/L acrolein for 6, 12, or 24 h. **A** Representative fluorescence images of mitotracker-labeled mitochondria showing the morphology (white squares in upper panels magnified in lower panels; scale bar, 20 μm). **B** Relative levels of mitochondrial ROS generation assessed by MitoSox Deep Red staining. **C** Mitochondrial membrane potential measured by TMRE staining. **D** Western blots (left) and analysis (right) of the levels of mitochondrial cytochrome c and cytoplasmic cytochrome c in each group. *n =* 6 per group in **A–D**. Data are presented as the mean ± SEM; *P < 0.05, **P < 0.01 *vs* vehicle group.

We next explored the possible changes in neurons after the accumulation of acrolein. First, primary neurons were treated with acrolein (25, 50, and 100 μmol/L) for 12 h. Mitotracker staining was used to evaluate the effects of acrolein on mitochondrial morphology. The results showed that, compared with the vehicle group, acrolein treatment evidently increased mitochondrial fragmentation in a dose-dependent manner (Fig. 2A). Then, a dose of 100 μmol/L was chosen to treat primary neurons at different time points (6, 12, and 24 h), which showed that acrolein increased the fragmentation in a time-dependent manner (Fig. 2A). Consistent with these results, mitochondrial functions were found to be impaired. MitoSOX and TMRE staining to evaluate mitochondrial ROS production and the mitochondrial MMP showed that acrolein increased the ROS production (Fig. 2B) and reduced the MMP (Fig. 2C) in the same manner as described above. Meanwhile, the release of cytochrome c (Cyt. c) from mitochondria to cytoplasm, which is a key step in mitochondrial apoptosis, was elevated after acrolein treatment both dose- and time-dependently (Fig. 2D). These results suggested that acrolein induces morphological alterations and the functional impairment of mitochondria in primary neurons.

### Inhibition of Drp1 Protects against Morphological Alterations and Functional Impairment of Mitochondria Induced by Acrolein

**Fig. 3:**
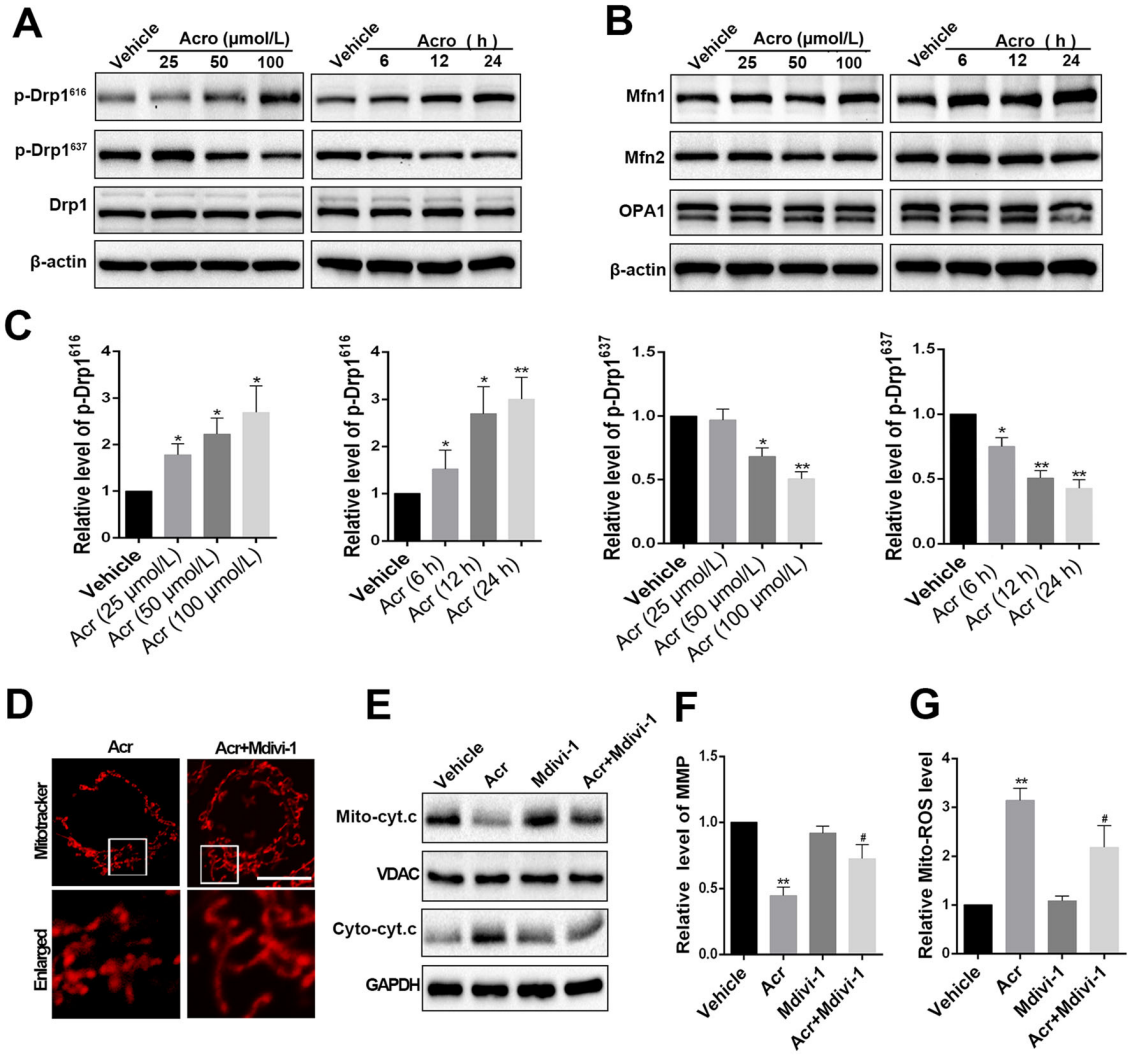
Drp1 contributes to the morphological alterations and functional impairment of mitochondria induced by acrolein. **A, C** Western blots and analysis of the levels of Drp1, p-Drp1^616^, and p-Drp1^637^ in each group. **B, C** Western blots and analysis of the levels of the mitochondrial fusion proteins Mfn1, Mfn2, and Opa1 in each group. **D** Typical images of the morphological characteristics of mitochondria labelled by Mitotracker in each group. (Scale bar, 20 μm; acrolein (100 μmol/L, 24 h); Mdivi-1 (10 μmol/L, 24 h). **E** Western blots showing the levels of mitochondrial and cytoplasmic cytochrome c in each group. **F** Mitochondrial membrane potential measured *via* TMRE staining. **G** Measurement of mitochondrial ROS generation *via* MitoSox Deep Red staining. Values are presented as the mean ± SEM, *n =* 6 per group. *P < 0.05, **P < 0.01 *vs* vehicle group; ^#^*P* < 0.05, ^##^*P* < 0.01 *vs* acrolein group.

The equilibrium of mitochondrial dynamics and normal mitochondrial morphology are critical for maintaining mitochondrial function. Since we found that acrolein induced mitochondrial fragmentation, we next identified key proteins that regulate mitochondrial fusion and fission. First, we focused on the modulator protein of mitochondrial fission, Drp1, which promotes mitochondrial fragmentation under multiple stresses by translocating from the cytoplasm to the mitochondrial membrane. The protein level of total Drp1 did not change significantly after acrolein treatment in mice. However, its phosphorylation level in the ICH group was markedly decreased at S637, while its phosphorylation at S616 was increased (Fig. 3A, C). As we mentioned above, previous studies demonstrated that phosphorylation of Drp1 at S616 accelerates its recruitment to the mitochondria membrane, while phosphorylation at S637 in Drp1 impairs this process. Therefore, the phosphorylation of Drp1 may be a key mechanism by which acrolein induces mitochondrial fragmentation. Meanwhile, we explored possible changes in the modulator proteins of mitochondrial fission Opa1, Mfn1 and Mfn2, whose low level might contribute to the impairment of mitochondrial integrity and the induction of fragmentation. The results showed that the levels of Opa1 and Mfn2 did not change significantly after acrolein treatment. And the Mfn1 was elevated with 100 μmol/L acrolein, which may be a consequence of compensation for the imbalance of mitochondrial dynamics (Fig. 3B, C). Then, in order to test the hypothesis that excessive mitochondrial translocation of Drp1 contributes to the acrolein-induced morphological alterations and functional impairment of mitochondria, we used the selective inhibitor of Drp1, Mdivi-1 (10 μmol/L, 24 h). The results showed that pharmacological inhibition of Drp1 reduced the induction of mitochondrial fragmentation (Fig. 3D) and the production of mitochondrial ROS (Fig. 3G), promoted restoration of the MMP (Fig. 3F), and inhibited the increased level of cytosolic cytochrome c (Fig. 3E) induced by acrolein (100 μmol/L, 24 h). These results suggested that Drp1-mediated excessive mitochondrial fission contributes to the morphological changes and functional impairment of mitochondria induced by acrolein.

### Acrolein Regulates the Mitochondrial Translocation of Drp1 Partly through AMPK and ERK Pathways

**Fig. 4:**
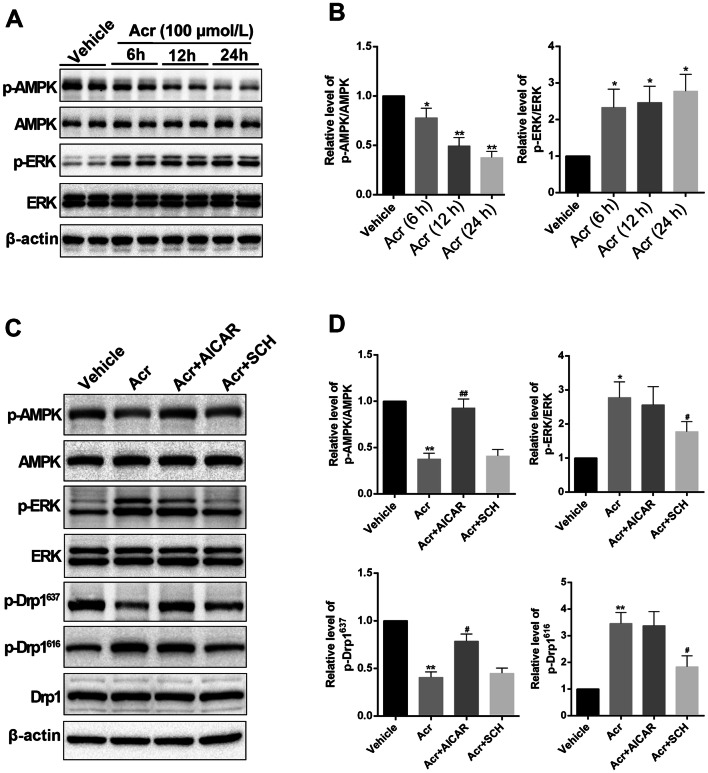
Acrolein regulates the translocation of Drp1 partly through the AMPK and ERK pathways. **A, B** Western blots (**A**) and analysis (**B**) of p-AMPK, AMPK, p-ERK, and ERK in different groups of primary neurons. **C, D** Western blots (**C**) and analysis (**D**) of p-AMPK, AMPK, p-ERK, ERK, p-Drp1^616^, and p-Drp1^637^ in different groups of primary neurons. Values are presented as the mean ± SEM, *n =* 6 per group. *P < 0.05, **P < 0.01 *vs* vehicle group. ^#^*P* < 0.05, ^##^*P* < 0.01 vs acrolein group.

The AMP-activated protein kinase (AMPK) and extracellular signal-regulated kinase (ERK) pathways are important kinases for the phosphorylation of Drp1 at S637 and S616, respectively [39–42]. In our study, acrolein (100 μmol/L) significantly decreased p-AMPK while increasing p-ERK in a time-dependent manner (Fig. 4A, B). Then, we found that the selective AMPK activator AICAR significantly reversed the decreased phosphorylation level of Drp1 at S637. And the selective inhibitor of the ERK pathway, SCH772984, markedly decreased the elevated phosphorylation level of Drp1 at S616 (Fig. 4C, D). All these data indicated that acrolein regulates the translocation of Drp1 from cytoplasm to mitochondria, at least partially, through the AMPK and ERK pathways.

### Hydralazine (Acrolein Scavenger) Suppresses the Translocation of Drp1 and Alleviates the Morphological Disruption of Mitochondria after ICH in Mice

**Fig. 5:**
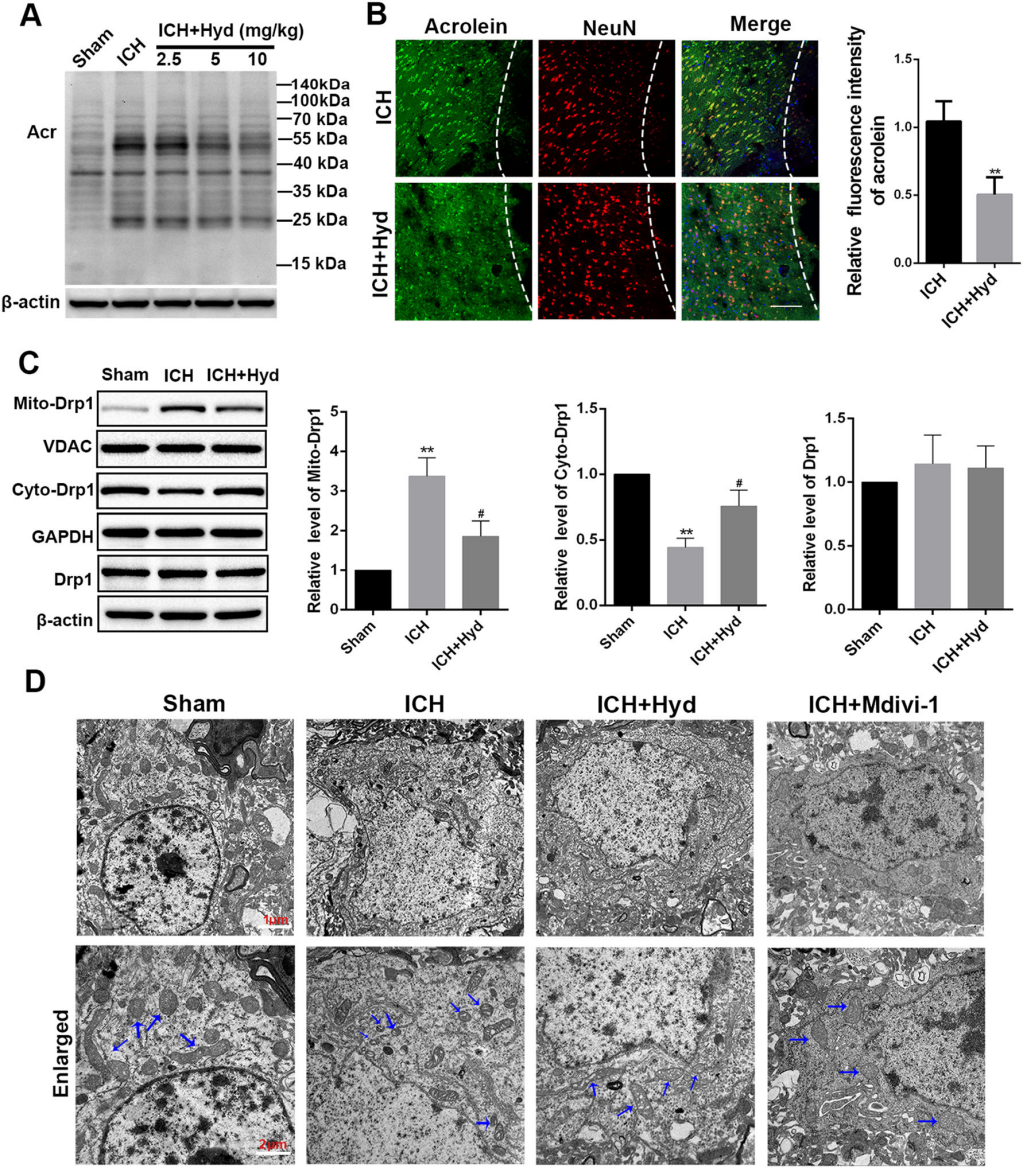
Hydralazine (acrolein scavenger) suppresses the translocation of Drp1 and alleviates the morphological disruption of mitochondria after ICH in mice. **A** Western blots showing the level of acrolein in each group (Hyd, hydralazine). **B** Representative images of double immunofluorescence staining for acrolein and NeuN in each group 72 h after operation (scale bar, 100 μm). **C** Western blots (left) and analysis (right) of cyto-Drp1, mito-Drp1, and T-Drp1 in different groups of mice. **D** Electron microscopic images showing the ultrastructural changes in neurons from mouse brain in each group (arrows represent mitochondria). Values are presented as the mean ± SEM, *n =* 6 per group, *P < 0.05, **P < 0.01 *vs* sham group, ^#^*P* < 0.05, ^##^*P* < 0.05 *vs* ICH group.

Based on the above evidence, we also hypothesized that elimination of acrolein could alleviate the Drp1-mediated morphological disruption of mitochondria after ICH. In order to further investigate the active role of acrolein in ICH, we explored the effects of hydralazine (an acrolein scavenger) on ICH injury in mice. We found that hydralazine effectively eliminated the level of acrolein in a concentration-dependent manner (Fig. 5A). Hydralazine at 10 mg/kg was chosen for further *in vivo* studies. Consistent with the above western blot results, the subsequent immunofluorescence analysis indicated that after additional hydralazine treatment (10 mg/kg), the relative fluorescence intensity of acrolein significantly declined, suggesting decreased levels of acrolein (Fig. 5B). Meanwhile, after the successful separation of mitochondria and cytosol (Fig. S2), the results showed that the mitochondrial level of Drp1 in the ICH group was markedly increased, while the cytosolic level of Drp1 was markedly decreased compared to the sham group (Fig. 5C). And these changes were reversed after hydralazine treatment (Fig. 5C). Then, we focused on the morphometric changes of mitochondria in each group. Normally, mitochondrial structure features a long tubular appearance with prominent cristae, as well as some small globular structures (Fig. 5D). However, ICH injury induced marked heterogeneity of mitochondria in size and shape. A large number of small, globular structures appeared, indicating mitochondrial fragmentation. In addition, the morphological abnormalities included swelling, collapsed cristae, and rupture of the mitochondrial membrane. However, hydralazine or Mdivi-1 treatment remarkably reduced the mitochondrial fragmentation as evidenced by increased tubular networks. These results indicated that the acrolein scavenger plays a protective role in the maintenance of mitochondrial morphology after ICH.

### Hydralazine Protects against Neural Apoptosis, Brain Edema, and Neurological Functional Deficits Following ICH in Mice

**Fig. 6:**
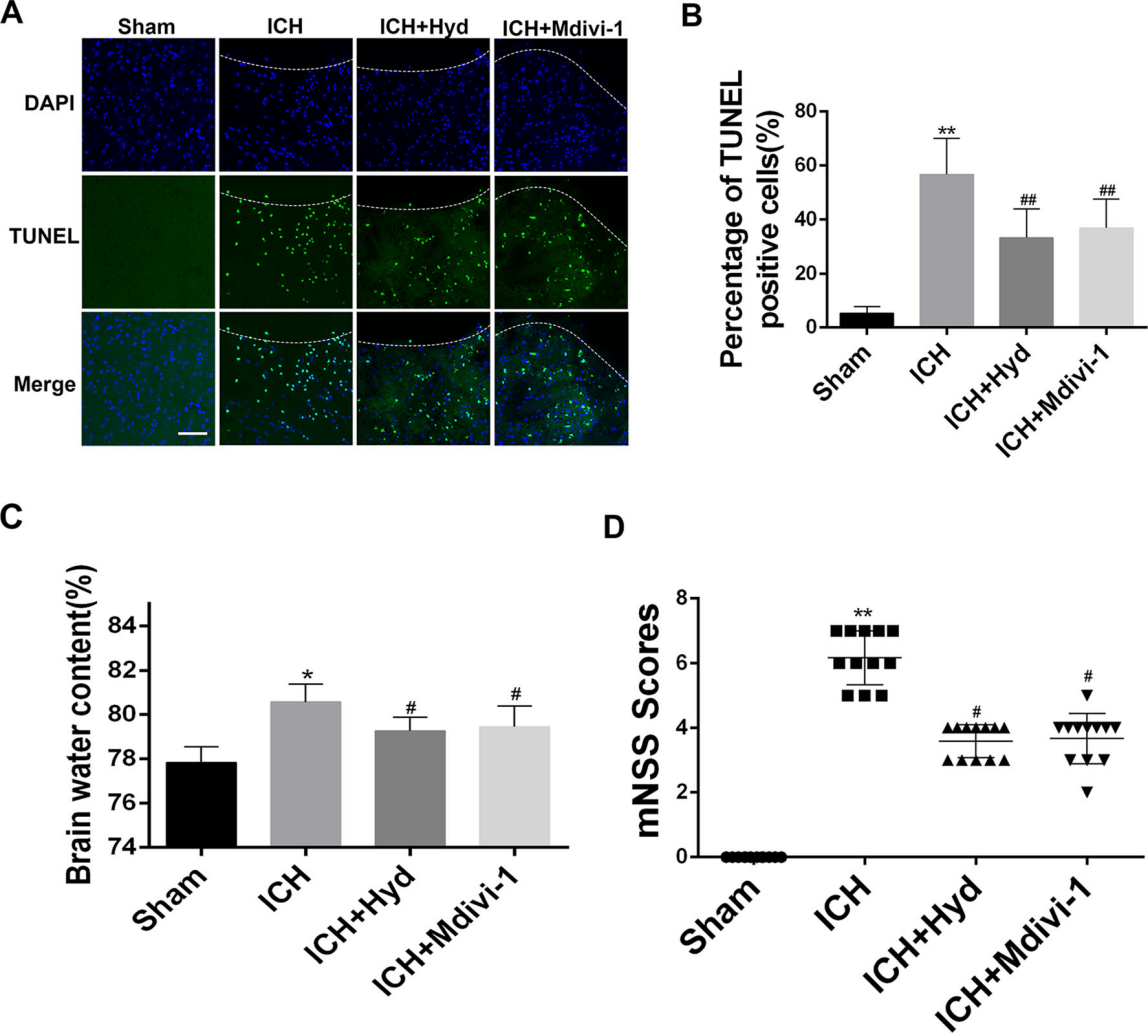
Effects of hydralazine (acrolein scavenger) on neural apoptosis, hematoma volume, brain water content, and neurological function scores in mice 72 h after ICH. Mdivi-1 treatment served as the positive control. **A, B** Representative TUNEL staining images (**A**) and analysis (**B**) in each group 72 h after operation (scale bar, 100 μm). **C** Brain water content in each group measured 72 h after ICH (*n* = 6). **D** Modified neurological severity scores (mNSS) in each group (*n* = 12). Values are presented as the mean ± SEM, *P < 0.05, **P < 0.01 *vs* sham group, ^#^*P* < 0.05, ^##^*P* < 0.01 *vs* ICH group.

To further explore the neuroprotective effects of acrolein scavenging on ICH injury, we measured neural apoptosis, hematoma volume, brain edema, and neurological deficits. Additional treatment of the ICH group with Mdivi-1 (a selective Drp1 inhibitor) was used as a positive control. TUNEL staining showed that additional hydralazine or Mdivi-1 treatment reduced the neural apoptosis after ICH (Fig. 6A, B). No significant differences in hematoma size among groups were observed (Fig. S3). However, although hydralazine or Mdivi-1 treatment was unable to reduce hematoma volume, it significantly alleviated the ICH-induced brain injury. We found that administration of hydralazine or Mdivi-1 significantly reduced the brain water content (Fig. 6C) and alleviated the neurological deficits (Fig. 6D) after ICH. All these results indicated that treatment with an acrolein scavenger may be a promising strategy to more effectively alleviate the neural apoptosis, brain edema, and neurological deficits after ICH in mice.

## Discussion

Our results suggest that acrolein, an unsaturated aldehyde, is a critical pathogenic factor in SBI after ICH. We found that acrolein induced mitochondrial fragmentation accompanied by loss of mitochondrial membrane potential, the generation of reactive oxidative species, and the release of mitochondrial cytochrome c. Further study indicated that the mitochondrial damage induced by acrolein is associated with increased mitochondrial Drp1 translocation and excessive mitochondrial fission. An acrolein scavenger significantly inhibited the Drp1-mediated fission and attenuated the morphological damage of mitochondria after ICH. Most importantly, neural apoptosis, brain edema, and neurological functional deficits were also significantly alleviated. Taken together, the study first proposes a critical role of acrolein in the pathological process of SBI following ICH, and underscores a new mechanism by which Drp1-mediated mitochondrial oxidative damage contributes to acrolein-induced neurotoxicity.

Cerebral stroke is a common acute event that can be divided into two categories, namely ischemic stroke and hemorrhagic stroke [43, 44]. The incomplete understanding of the molecular mechanisms that contribute to SBI has restricted clinical advances in ICH treatment. Although potential treatments for SBI have been proposed, their effects on ICH sufferers have usually failed to live up to expectations. So we speculated that there may be pivotal pathological factors in SBI that have not been noted previously. A neurotoxic role of acrolein has been indicated in the pathological process of neurodegenerative diseases, traumatic spinal cord injury, and ischemic stroke [15–17]. In our study, we found a significant increase in acrolein after ICH, which inspired us to explore whether acrolein plays a causal role in SBI following ICH. Mitochondria are cell-death executors and mitochondrial quality control plays an essential role in protecting against ICH injury. We found that acrolein treatment induced the impairment of mitochondrial morphology and the failure of mitochondrial function in primary neuronal cultures. Although acrolein is known to result in mitochondrial dysfunction *via* a variety of intra- and inter-cellular signaling mechanisms, few studies have focused on the role of mitochondrial dynamics in acrolein-induced damage, especially in acrolein-induced neural injury.

Since we found that acrolein induced mitochondrial fragmentation and swelling, we next determined the key proteins that regulate mitochondrial fusion and fission. The results showed that the recruitment of Drp1 to mitochondria (an indispensable step for mitochondrial fission) was significantly enhanced. This indicated that acrolein promotes neural apoptosis by changing the balance of mitochondrial fission and fusion proteins toward fission. Previous studies have demonstrated that excessive mitochondrial fission contributes to mitochondrial fragmentation, evokes oxidative cellular injury, impairs mitochondrial energy production, and facilitates the mitochondrial apoptosis pathway. Here, we found that the acrolein-induced mitochondrial fragmentation, mitochondrial ROS production, MMP impairment, and cytochrome c release were suppressed by Mdivi-1, a selective inhibitor of Drp1. These results from pharmacological inhibition of Drp1 suggest that Drp1-mediated mitochondrial fission plays a critical role in acrolein-induced mitochondrial damage and neural injury. It is generally accepted at present that phosphorylation of Drp1 at Ser616 accelerates its recruitment to mitochondrial membrane, while phosphorylation at Ser637 inhibits this process. We then found that the regulation of Drp1 translocation by acrolein, at least in part, relied on AMPK and ERK pathways which targeted the phosphorylation of Drp1 at Ser637 and Ser616, respectively. However, the post-translational modifications of Drp1 which regulate its recruitment to mitochondria are a complicated process, including not only phosphorylation, but also S-nitrosilation, ubiquitination, and sumoylation [27, 45, 46]. Therefore, broader mechanisms underlying the process deserve further study.

In order to confirm the critical role of acrolein in ICH injury and to explore whether its elimination can alleviate the SBI following ICH insults, we treated ICH mice with a acrolein scavenger, hydralazine. Surprisingly, we found that hydralazine mitigated the acrolein-induced mitochondrial translocation of Drp1 and the morphological disruption of mitochondria, resulting in significant amelioration of neural apoptosis, brain edema, and neurological deficits. These results not only confirmed the pathological role of acrolein in SBI after ICH, but also suggested a neuroprotective role of acrolein scavengers in the treatment of ICH. In view of the extensive participation of acrolein in various disorders, elaborating the relevant mechanism and developing acrolein-targeting strategies are of vital importance for future clinical treatment.

In this study, we mainly studied the role acrolein plays in the early brain injury after ICH, while its role in late-stage injury is not yet clear. Previous studies reported that acrolein contributes to the impairment of cognitive functions in cerebral degenerative diseases [15]. So whether acrolein plays a critical role in long-term cognitive dysfunction in ICH patients deserves further research. Meanwhile, an increased plasma level of acrolein is a good biomarker for brain infarction in cerebral ischemic stroke patients [17]. This inspired us to explore whether the plasma level of acrolein is also a good biomarker for ICH and for the guidance of future clinical treatment. This tool for the early identification of ICH may help in the application of suitable therapy for ICH suffers.

There is another point worth noting. Normal Drp1-mediated fission is of vital importance in the removal of impaired mitochondria to maintain mitochondrial homeostasis, while excessive fission impairs mitochondrial structure, leading to damaged respiratory function, increased mitochondrial ROS generation, a shortage of ATP, and the activation of apoptosis pathways [39, 47]. Previous studies have shown that the Drp1 inhibitor, Mdivi-1, ameliorates oxidative stress and neural apoptosis after subarachnoid hemorrhage [22, 48]. In our study, we also showed the neuroprotective effects of inhibition of Drp1 with Mdivi-1 in an ICH model (Figures 5 and 6), suggesting that excessive Drp1 activity may be a crucial pathogenic factor in ICH-induced brain injury. Meanwhile, there are lines of evidence indicating the benefits of mitochondrial fragmentation; the fragmented mitochondria are prone to be eliminated by autophagosomes, thus protecting the cells from death. Given the different disease models and different pathological processes, whether Drp1-mediated fragments contribute to autophagy and thus promote neural survival after ICH is not clear. Notably, autophagy may not only block the induction of apoptosis, but excessive autophagy also helps to induce apoptosis and aggravates neural injury [49]. Our current study may not provide a definite answer to explain the potential relationship between Drp1 and autophagy after ICH, but we believe that it points to exciting avenues for future research, and it is also worth extensive and intensive discussion.

In conclusion, we describe the crucial and complicated role of acrolein in ICH injury and underscore a new mechanism that Drp1-mediated mitochondrial fission is involved in the neurotoxicity of acrolein. Moreover, we suggest that acrolein scavenging may have promising clinical applications and greatly benefit ICH sufferers.

## Electronic supplementary material

Below is the link to the electronic supplementary material.

Supplementary material 1 (PDF 1351 kb)

## References

[1] Al-Shahi Salman R, Frantzias J, Lee RJ, Lyden PD, Battey TWK, Ayres AM (2018). Absolute risk and predictors of the growth of acute spontaneous intracerebral haemorrhage: a systematic review and meta-analysis of individual patient data. Lancet Neurol.

[2] Biffi A, Anderson CD, Battey TW, Ayres AM, Greenberg SM, Viswanathan A (2015). Association between blood pressure control and risk of recurrent intracerebral hemorrhage. JAMA.

[3] Wang Z, Zhou F, Dou Y, Tian X, Liu C, Li H (2018). Melatonin alleviates intracerebral hemorrhage-induced secondary brain injury in rats via suppressing apoptosis, inflammation, oxidative stress, DNA damage, and mitochondria injury. Transl Stroke Res.

[4] Han N, Ding SJ, Wu T, Zhu YL (2008). Correlation of free radical level and apoptosis after intracerebral hemorrhage in rats. Neurosci Bull.

[5] Zhu H, Wang Z, Yu J, Yang X, He F, Liu Z (2019). Role and mechanisms of cytokines in the secondary brain injury after intracerebral hemorrhage. Prog Neurobiol.

[6] Lan X, Han X, Li Q, Yang QW, Wang J (2017). Modulators of microglial activation and polarization after intracerebral haemorrhage. Nat Rev Neurol.

[7] Zhang P, Wang T, Zhang D, Zhang Z, Yuan S, Zhang J (2019). Exploration of MST1-mediated secondary brain injury induced by intracerebral hemorrhage in rats via hippo signaling pathway. Transl Stroke Res.

[8] Li YJ, Chang GQ, Liu Y, Gong Y, Yang C, Wood K (2015). Fingolimod alters inflammatory mediators and vascular permeability in intracerebral hemorrhage. Neurosci Bull.

[9] Esterbauer H, Schaur RJ, Zollner H (1991). Chemistry and biochemistry of 4-hydroxynonenal, malonaldehyde and related aldehydes. Free Radic Biol Med.

[10] Burcham PC, Fontaine FR, Kaminskas LM, Petersen DR, Pyke SM (2004). Protein adduct-trapping by hydrazinophthalazine drugs: mechanisms of cytoprotection against acrolein-mediated toxicity. Mol Pharmacol.

[11] Igarashi K, Uemura T, Kashiwagi K (2018). Acrolein toxicity at advanced age: present and future. Amino Acids.

[12] Luo J, Robinson JP, Shi R (2005). Acrolein-induced cell death in PC12 cells: role of mitochondria-mediated oxidative stress. Neurochem Int.

[13] Liu-Snyder P, McNally H, Shi R, Borgens RB (2006). Acrolein-mediated mechanisms of neuronal death. J Neurosci Res.

[14] Tully M, Zheng L, Acosta G, Tian R, Shi R (2014). Acute systemic accumulation of acrolein in mice by inhalation at a concentration similar to that in cigarette smoke. Neurosci Bull.

[15] Shamoto-Nagai M, Maruyama W, Hashizume Y, Yoshida M, Osawa T, Riederer P (2007). In parkinsonian substantia nigra, alpha-synuclein is modified by acrolein, a lipid-peroxidation product, and accumulates in the dopamine neurons with inhibition of proteasome activity. J Neural Transm (Vienna).

[16] Park J, Muratori B, Shi R (2014). Acrolein as a novel therapeutic target for motor and sensory deficits in spinal cord injury. Neural Regen Res.

[17] Saiki R, Nishimura K, Ishii I, Omura T, Okuyama S, Kashiwagi K (2009). Intense correlation between brain infarction and protein-conjugated acrolein. Stroke.

[18] Zheng J, Shi L, Liang F, Xu W, Li T, Gao L (2018). Sirt3 ameliorates oxidative stress and mitochondrial dysfunction after intracerebral hemorrhage in diabetic rats. Front Neurosci.

[19] Zhou Y, Wang S, Li Y, Yu S, Zhao Y (2017). SIRT1/PGC-1alpha signaling promotes mitochondrial functional recovery and reduces apoptosis after intracerebral hemorrhage in rats. Front Mol Neurosci.

[20] Lee JE, Westrate LM, Wu H, Page C, Voeltz GK (2016). Multiple dynamin family members collaborate to drive mitochondrial division. Nature.

[21] Franco A, Kitsis RN, Fleischer JA, Gavathiotis E, Kornfeld OS, Gong G (2016). Correcting mitochondrial fusion by manipulating mitofusin conformations. Nature.

[22] Fan LF, He PY, Peng YC, Du QH, Ma YJ, Jin JX (2017). Mdivi-1 ameliorates early brain injury after subarachnoid hemorrhage via the suppression of inflammation-related blood-brain barrier disruption and endoplasmic reticulum stress-based apoptosis. Free Radic Biol Med.

[23] Zhang T, Wu P, Zhang JH, Li Y, Xu S, Wang C (2018). Docosahexaenoic acid alleviates oxidative stress-based apoptosis via improving mitochondrial dynamics in early brain injury after subarachnoid hemorrhage. Cell Mol Neurobiol.

[24] Wang X, Jiang W, Yan Y, Gong T, Han J, Tian Z (2014). RNA viruses promote activation of the NLRP3 inflammasome through a RIP1-RIP3-DRP1 signaling pathway. Nat Immunol.

[25] Xu M, Wang L, Wang M, Wang H, Zhang H, Chen Y (2019). Mitochondrial ROS and NLRP3 inflammasome in acute ozone-induced murine model of airway inflammation and bronchial hyperresponsiveness. Free Radic Res.

[26] Song M, Dorn GW (2015). Mitoconfusion: noncanonical functioning of dynamism factors in static mitochondria of the heart. Cell Metab.

[27] Prieto J, Leon M, Ponsoda X, Sendra R, Bort R, Ferrer-Lorente R (2016). Early ERK1/2 activation promotes DRP1-dependent mitochondrial fission necessary for cell reprogramming. Nat Commun.

[28] Wikstrom JD, Israeli T, Bachar-Wikstrom E, Swisa A, Ariav Y, Waiss M (2013). AMPK regulates ER morphology and function in stressed pancreatic beta-cells via phosphorylation of DRP1. Mol Endocrinol.

[29] Cereghetti GM, Stangherlin A, Martins de Brito O, Chang CR, Blackstone C, Bernardi P*, et al.* Dephosphorylation by calcineurin regulates translocation of Drp1 to mitochondria. Proc Natl Acad Sci U S A 2008, 105: 15803–15808. 10.1073/pnas.0808249105 PMC2572940 18838687

[30] Aydin B, Atli Sekeroglu Z, Sekeroglu V (2018). Effects of whey protein and conjugated linoleic acid on acrolein-induced cardiac oxidative stress, mitochondrial dysfunction and dyslipidemia in rats. Biomed Pharmacother.

[31] Fang Y, Tian Y, Huang Q, Wan Y, Xu L, Wang W (2019). Deficiency of TREK-1 potassium channel exacerbates blood-brain barrier damage and neuroinflammation after intracerebral hemorrhage in mice. J Neuroinflammation.

[32] Rios JA, Godoy JA, Inestrosa NC (2018). Wnt3a ligand facilitates autophagy in hippocampal neurons by modulating a novel GSK-3beta-AMPK axis. Cell Commun Signal.

[33] Xi Y, Feng D, Tao K, Wang R, Shi Y, Qin H (2018). MitoQ protects dopaminergic neurons in a 6-OHDA induced PD model by enhancing Mfn2-dependent mitochondrial fusion via activation of PGC-1alpha. Biochim Biophys Acta Mol Basis Dis.

[34] Liu H, Zhao L, Yue L, Wang B, Li X, Guo H (2017). Pterostilbene attenuates early brain injury following subarachnoid hemorrhage via inhibition of the NLRP3 inflammasome and Nox2-related oxidative stress. Mol Neurobiol.

[35] Chen J, Li Y, Wang L, Zhang Z, Lu D, Lu M (2001). Therapeutic benefit of intravenous administration of bone marrow stromal cells after cerebral ischemia in rats. Stroke.

[36] Zhao H, Zhang K, Tang R, Meng H, Zou Y, Wu P (2018). TRPV4 blockade preserves the blood-brain barrier by inhibiting stress fiber formation in a rat model of intracerebral hemorrhage. Front Mol Neurosci.

[37] Li X, Guo H, Zhao L, Wang B, Liu H, Yue L (2017). Adiponectin attenuates NADPH oxidase-mediated oxidative stress and neuronal damage induced by cerebral ischemia-reperfusion injury. Biochim Biophys Acta Mol Basis Dis.

[38] Fang Z, Feng Y, Li Y, Deng J, Nie H, Yang Q (2019). Neuroprotective autophagic flux induced by hyperbaric oxygen preconditioning is mediated by cystatin C. Neurosci Bull.

[39] Zhou H, Wang S, Zhu P, Hu S, Chen Y, Ren J (2018). Empagliflozin rescues diabetic myocardial microvascular injury via AMPK-mediated inhibition of mitochondrial fission. Redox Biol.

[40] Li J, Wang Y, Wang Y, Wen X, Ma XN, Chen W (2015). Pharmacological activation of AMPK prevents Drp1-mediated mitochondrial fission and alleviates endoplasmic reticulum stress-associated endothelial dysfunction. J Mol Cell Cardiol.

[41] Huang CY, Chiang SF, Chen WT, Ke TW, Chen TW, You YS (2018). HMGB1 promotes ERK-mediated mitochondrial Drp1 phosphorylation for chemoresistance through RAGE in colorectal cancer. Cell Death Dis.

[42] Huang CY, Lai CH, Kuo CH, Chiang SF, Pai PY, Lin JY (2018). Inhibition of ERK-Drp1 signaling and mitochondria fragmentation alleviates IGF-IIR-induced mitochondria dysfunction during heart failure. J Mol Cell Cardiol.

[43] Liang S, Jiang X, Zhang Q, Duan S, Zhang T, Huang Q (2018). Abnormal metabolic connectivity in rats at the acute stage of ischemic stroke. Neurosci Bull.

[44] Hu HM, Li B, Wang XD, Guo YS, Hui H, Zhang HP (2018). Fluoxetine is neuroprotective in early brain injury via its anti-inflammatory and anti-apoptotic effects in a rat experimental subarachnoid hemorrhage model. Neurosci Bull.

[45] Otera H, Ishihara N, Mihara K (2013). New insights into the function and regulation of mitochondrial fission. Biochim Biophys Acta.

[46] Chan DC (2012). Fusion and fission: interlinked processes critical for mitochondrial health. Annu Rev Genet.

[47] Yang X, Wang H, Ni HM, Xiong A, Wang Z, Sesaki H (2017). Inhibition of Drp1 protects against senecionine-induced mitochondria-mediated apoptosis in primary hepatocytes and in mice. Redox Biol.

[48] Wu P, Li Y, Zhu S, Wang C, Dai J, Zhang G (2017). Mdivi-1 alleviates early brain injury after experimental subarachnoid hemorrhage in rats, possibly via inhibition of drp1-activated mitochondrial fission and oxidative stress. Neurochem Res.

[49] Song S, Tan J, Miao Y, Li M, Zhang Q (2017). Crosstalk of autophagy and apoptosis: Involvement of the dual role of autophagy under ER stress. J Cell Physiol.

