# Supplementary material for: Acrolein Aggravates Secondary Brain Injury After Intracerebral Hemorrhage Through Drp1-Mediated Mitochondrial Oxidative Damage in Mice
Source: Neurosci Bull. 2020 May 21;36(10):1158–70. doi: 10.1007/s12264-020-00505-7 (PMC7532238; doi:10.1007/s12264-020-00505-7)
Supplement: Supplementary file 1 — Supplementary material 1 (PDF 1351 kb) [file 12264_2020_505_MOESM1_ESM.pdf]

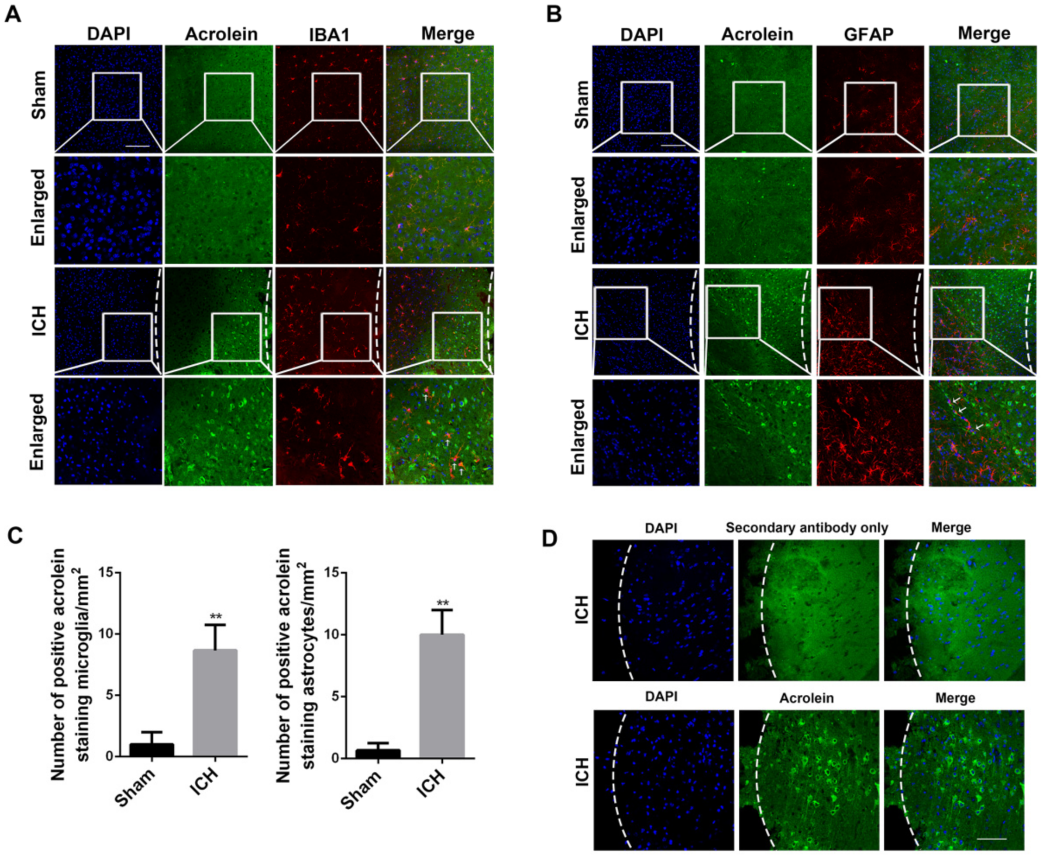

Fig. S1 A, B Representative images of double immunofluorescence staining for acrolein (green) with IBA1 (red in A) or GFAP (red in B) in the peri-hematomal area 72 h after ICH (arrows, acrolein-positive cells; dashed lines, hemorrhage sites). C Semi-quantification of acrolein-positive microglia and astrocytes *(n* = 6 mice per group; data are presented as the mean ± SEM; ***P* < 0.01 *vs* sham group). **D** Representative images of ICH mouse brain sections incubated with secondary antibodies only as a negative control to demonstrate the specificity of acrolein antibody (scale bar, 200 μm).

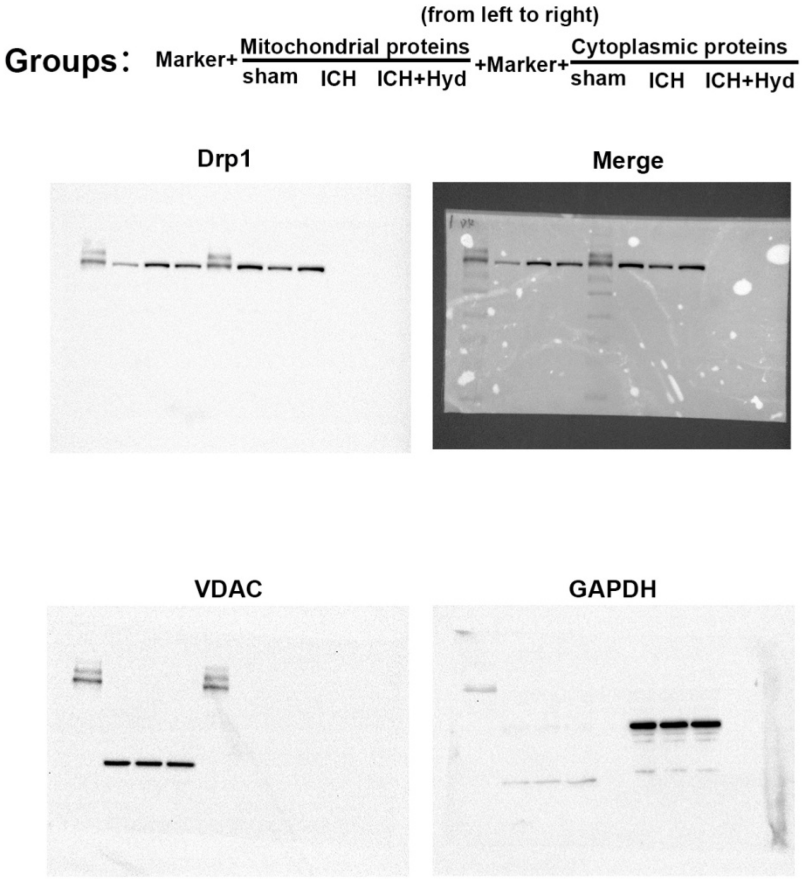

Fig. S2 Original images of protein bands of cyto-Drp1, mito-Drp1, Drp1, VDAC, and GAPDH in different groups of mice.

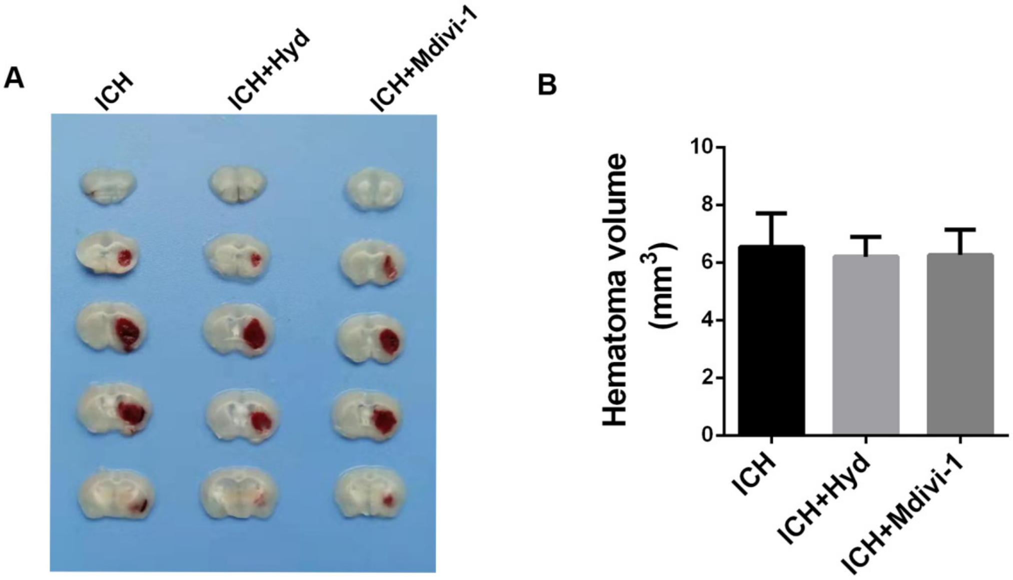

Fig. S3 **A** Typical images of brain slices from mice 72 h after ICH (red, hematoma area). **B** Statistical analysis of hematoma volumes in brain slices using ImageJ software (n = 6 per group).
